# A Novel Videography Method for Generating Crack-Extension Resistance Curves in Small Bone Samples

**DOI:** 10.1371/journal.pone.0055641

**Published:** 2013-02-06

**Authors:** Orestis L. Katsamenis, Thomas Jenkins, Federico Quinci, Sofia Michopoulou, Ian Sinclair, Philipp J. Thurner

**Affiliations:** 1 Bioengineering Sciences Research Group, Faculty of Engineering and the Environment, University of Southampton, Southampton, United Kingdom; 2 National Centre for Advanced Tribology at Southampton (nCATS), Faculty of Engineering and the Environment, University of Southampton, Southampton, United Kingdom; 3 Institute of Nuclear Medicine, University College London Hospitals NHS Foundation Trust, London, United Kingdom; 4 Engineering Materials Research Group, Faculty of Engineering and the Environment, University of Southampton, Southampton, United Kingdom; University of Notre Dame, United States of America

## Abstract

Assessment of bone quality is an emerging solution for quantifying the effects of bone pathology or treatment. Perhaps one of the most important parameters characterising bone quality is the toughness behaviour of bone. Particularly, fracture toughness, is becoming a popular means for evaluating bone quality. The method is moving from a single value approach that models bone as a linear-elastic material (using the stress intensity factor, K) towards full crack extension resistance curves (R-curves) using a non-linear model (the strain energy release rate in J-R curves). However, for explanted human bone or small animal bones, there are difficulties in measuring crack-extension resistance curves due to size constraints at the millimetre and sub-millimetre scale. This research proposes a novel “whitening front tracking” method that uses videography to generate full fracture resistance curves in small bone samples where crack propagation cannot typically be observed. Here we present this method on sharp edge notched samples (<1 mm×1 mm×Length) prepared from four human femora tested in three-point bending. Each sample was loaded in a mechanical tester with the crack propagation recorded using videography and analysed using an algorithm to track the whitening (damage) zone. Using the “whitening front tracking” method, full R-curves and J-R curves could be generated for these samples. The curves for this antiplane longitudinal orientation were similar to those found in the literature, being between the published longitudinal and transverse orientations. The proposed technique shows the ability to generate full “crack” extension resistance curves by tracking the whitening front propagation to overcome the small size limitations and the single value approach.

## Introduction

The interest in measuring fracture toughness behaviour of bone tissue is increasing within the bone research community as it is a quantitative way to evaluate an important bone quality parameter. Fracture toughness measurement techniques have been used in an increasing number of studies to quantify the fracture resistance of bone [1], [2], [3], [4], [5], [6], [7], [8]. These studies were able to provide a good estimate of bone fracture toughness in terms of the critical stress intensity factor (*K_c_*) and/or the critical strain energy release rate (*J-Integral*) while they were also pushing fracture toughness testing to the limits; in many cases, samples only a few millimetres in size were investigated due to size and shape constraints of available tissue samples (Table 1) [1], [2], [3], [4], [5], [6], [7], [8].

**Table1:** Sample sizes and geometries used by other researchers.

Authors	Sample	Thickness (mm)	Width (mm)	Length* (mm)	Year	Reference
T. L. Norman	CT	3,5,7 and 9	17.5	16.8	1995	[30]
D. Vashishth	CT	3	14	16.8	1997	[15]
P. Zioupos	3-point bending	4	4	*16	1998	[1]
J. B. Phelps	3-point bending	2	4	16	2000	[2]
	sandwich - CT	2	3.5	21		
X Wang	3-point bending	2	4	30	2002	[3]
C.Malik	CT	5	20.32	*80	2003	[4]
D. Vashishth	CT		14		2004	[5]
R.Nalla	CT	1.2–3.3	13–18.3	52–73	2004	[6]
J Yan	3-point bending	4	4	45 and 25	2007	[7]
E. Zimmermann	4-point bending	2.0–3.4	3.1–4.9	*12.4–19.6	2010	[8]

* if not reported is calculated as 4*W.

Beyond these experiments there is a further need for experimental methods to measure fracture toughness in even smaller samples to allow the quantification of bone fragility in a larger range of human and animal bones. In addition, bone tissue fracture toughness behaviour is likely to differ with sample size, due to the predominance of different hierarchical structures or defects at different sample sizes (the size effect). Evaluation of the “size effect” in fracture toughness may be crucial for understanding the contribution of different hierarchical levels to the ultimate fracture resistance of bone. Likewise, fracture toughness of individual human trabeculae (typical dimensions: length 2–4 mm; diameter 0.2–0.5 mm) has so far not been carried out as, until now, no technique has been available to measure fracture toughness in a sample of this small size. Fracture mechanics are not directly applicable on samples where the microstructural features are less than an order of magnitude smaller than the critical dimensions of the samples (i.e., the crack length and sample width). However, such measurements could be used for studying relative differences between single trabeculae or other small scale samples and so could still provide valuable information about their toughness. Furthermore, small-animal models (i.e. rat or mouse) are often used to study the effects of various factors on bone quality, such as disease, pharmaceutical treatment and genetic or epigenetic predisposition to bone disease. However, because of the small dimensions e.g. the femur of small rodent mammals (rats: 30–40 mm long and 3–4 mm diameter; mice: ∼15 mm long and 1–2 mm diameter), generating a crack resistance curve (R-curve) is very difficult and generally only a single-value *K_c_* is measured instead [9].

The experimental procedure for determining the crack-extension resistance (R-curve or J-R curve) of a material requires the measurement of crack extension (*Δa; Δa = a_(i)_ – a_0_*) that occurs during loading of a pre-cracked specimen [10]. In the case of sub-millimetre sized samples this is a very difficult task. To deal with this problem, two studies have used *in-situ* environmental scanning electron microscopy (ESEM) during three-point bending but these, to the best of our knowledge, were the only studies able to generate crack extension resistance curves for short (<600 µm) crack propagations [11], [12].

Here we present a new method for generating crack extension resistance curves in notched small bone samples, i.e. with cross-sectional dimensions of 1 mm×1 mm or less, tested in three-point bending combined with videography. Our approach is based on tracking the crack-front propagation via monitoring of the so-called “whitening zone”, which develops in front of the crack-tip. This approach tracks the actual crack propagation in an indirect fashion. Micro-cracking, is one of the intrinsic toughening mechanisms acting in front of the crack-tip during crack propagation in quasi-brittle materials like bone [13], [14], [15]. The “whitening effect” is the result of increased light reflection on the surfaces of the newly formed microcracks within this damage zone [16]. As the strain increases, some of the microcrack of the damage zone (also called frontal process zone [15]) are joint together and the main crack propagates. Subsequently, a new frontal process zone develops ahead of the propagated crack-tip and the process continues until the specimen fails [13], [15].

The main objectives of this study were (i) to quantify the correlation between the “whitening effect” and the crack propagation and (ii) to develop a computer-aided methodology to generate crack-propagation resistance curves for fracture toughness evaluation of small bone specimens.

## Materials and Methods

This study has been approved by the NHS, Health Research Authority. NRES Committee South Central - Southampton A. REC reference: 12/SC/0325.

### 2.1 Specimen Preparation and Testing

#### 2.1.1 Rat tibiae (whole bone) samples

Whole rat tibiae were used to access the applicability of the method on small animal model studies. For this purpose, two whole tibiae were harvested from 28 day old rats. At this age, the tibia is approximately 2–3 mm in diameter and about 20–25 mm long. After removal of soft tissue using tissue tweezers and a scalpel, bones were mounted on a low-speed saw and both the distal and proximal ends were removed. Subsequently, bone marrow was removed using a water-jet, and finally the posterior surface of the midshaft was notched using; firstly the low-speed saw (IsoMet, Buehler, Lake Bluff, IL, USA) and secondly a razor blade and diamond suspension, as described by Kruzic et al [17]. The pre-notched samples were loaded in a three-point bending rig with a 10 mm span submerged in Hanks’ Balanced Salt Solution (HBSS) of pH ≈ 7.4. Force was applied at 0.01 mm/s to failure by the mechanical tester (ElectroForce3200, Bose, Eden Prairie, MN, USA) with the posterior, notched surface of the bone in tension. Crack propagation was recorded using a high-speed camera (Ultima 512, Photron, San Diego, CA, USA) operated at 60 fps with two fiber optic lights (DC-950H, Dolan-Jenner, Boxborough, MA, USA) illuminating the specimen from approximately +45° and –45° from the camera field axis. The camera started recording in synchrony with the loading test initiation by the use of an external trigger. During the experiment “Force - Displacement” and “Force - Elapsed Time” channels were recorded. The “Force - Elapsed Time” channel was used to synchronize the high-speed video with the “Force - Displacement” channel using the Force as the reference point between the two channels. In more detail, as bending and videography experiments started simultaneously, the first point of the Force - Displacement curve (F_0_−v_0_) corresponds to the first point of the Force - Elapsed Time (t_0_−F_0_). Thus, Frame(0) corresponds to F_0_−v_0_. Consequently, knowing the recording frame rate (i.e. 60fps) the elapsed time of the random Frame(X) is X/60 second. Having calculated the time and using Force as reference point, the exact point on the Force - Displacement curve that a recorded event happened can be defined as follows: t_X/60_ corresponds to the force F_x_ on the “Force - Elapsed Time” curve (i.e. F_X/60_) which in turn corresponds to v_X/60_ on the “Force - Displacement” curve. This way Frame(X) is associated with the corresponding F and v of the event.

#### 2.1.2 Human cortical bone samples

**Figure 1:**
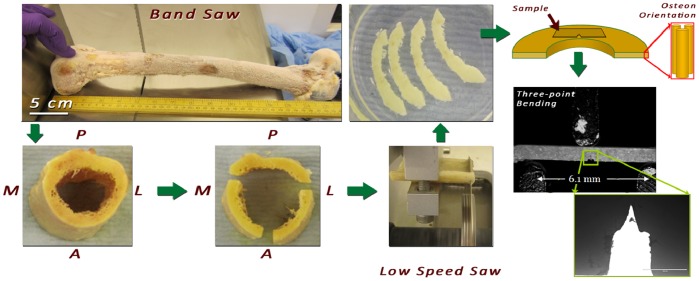
Miniature SE(B) sample preparation from a human femur.

Four human femora (females; aged 43, 47, 80 and 83) were obtained from the International Institute for the Advancement of Medicine (IIAM) and stored at −80°C. A butcher’s bandsaw (BG 200, Medoc, Logrono, Spain) and a low speed precision saw were used for cutting the femora into single-edge notched three-point bend SE(B) specimens [10] (n = 10) of 0.8–0.9 mm width and height and 10 mm length oriented in the antiplane longitudinal orientation [18] (Figure 1). Toughness experiments were conducted in three-point bending as described above except that a bending rig with a span of 6.15 mm instead of 10 mm was used due to sample size limitations. The tests were conducted using fully hydrated samples in air. To ensure hydration (i) all samples were submerged in HBSS for four (4) hours before the experiments and only removed just prior to testing (ii) testing time was kept in all cases to less than 1 minute to prevent sample dehydration.

### 2.2 Whitening-front Propagation Tracking

#### 2.2.1 Calibration and pre-processing

To reduce the computational power required, one in every 15 frames were sampled from the captured video. After loading the frames and load-time-displacement data, the first video frame was used for interactive pixel size calibration. This was achieved by selecting the contact points of the bottom (left and right) supports to the sample surface. The distance between these points corresponds to a known distance of 6.15 mm (cf. Figure 1) and this process allows for calculation of displacement values independent of the camera to sample distance. Next, a rectangular region of interest (ROI) surrounding the sample notch was selected for processing. Gamma correction and windowing was applied for video pre-processing aiming to standardize the image appearance across the different frames.

#### 2.2.2 Registration and subtraction

A subtraction method was used to enhance the whitening effect on the video. This method works on a frame-by-frame basis. First the initial frame is registered to the current frame using a normalized cross-correlation method introduced by Guizar et al. [19]. This is a rigid registration method which provides sub-pixel image registration without deforming the sample geometry. Next the difference image was calculated by subtracting the registered initial frame from the current frame. The same process was then repeated for all frames and the propagation of whitening across the sample can be seen in the resulting subtraction video (Videos S1, Video S2, Video S3).

#### 2.2.3 Whitening front propagation

**Figure 2:**
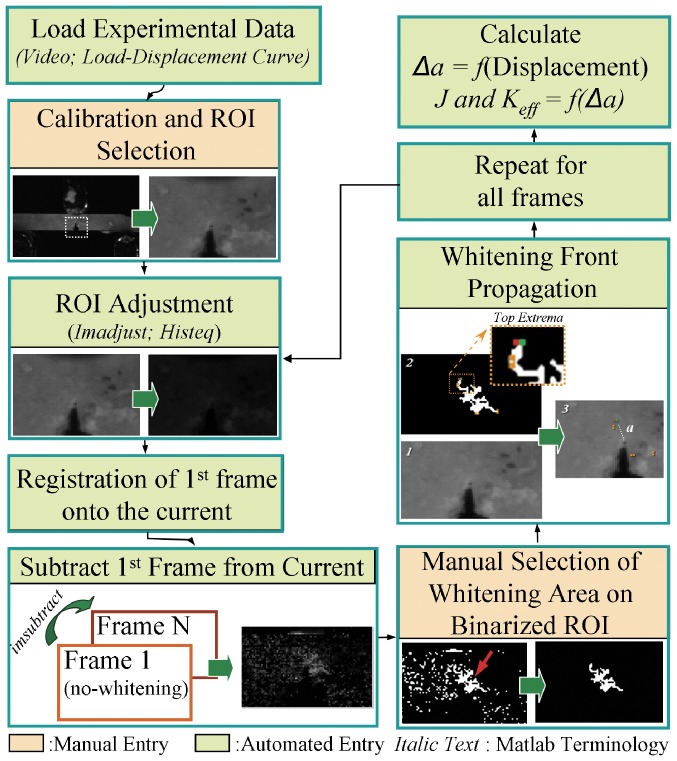
Outline of the “Whitening Front Tracking” algorithm.

**Figure 3:**
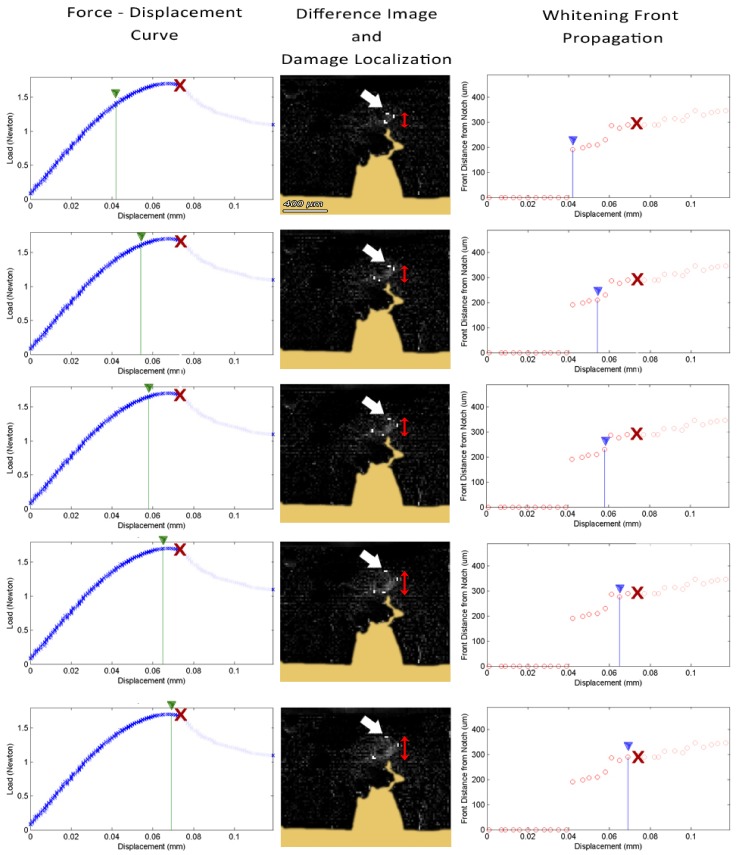
Time-lapsed snapshots of the fracture toughness experiment on an SE(B) sample also presented in video S2. (left) Force – Displacement curve; (middle) damage localization on the calculated difference image; (right) Calculated whitening front propagation –top-most white localiser pixel pointed by the white arrow on damage localization picture– red X represents point of failure.

The whitening front for each frame was automatically calculated from the corresponding difference image. The whitening region was identified by thresholding, while morphological opening with a structural element of 2 pixels in diameter was used to join neighbouring whitening regions together by removing small dark islands. The whitening-front was defined as the maximum of the top-left and top-right extrema of the whitening region. The front displacement was calculated as the distance between the whitening-front and the initial tip of the notch. The whitening-front propagation tracking algorithm is outlined in Figure 2 and an example dataset showing the whitening progression is presented in Figure 3.

### 2.3 Quantification of Correlation between Whitening Front- and Crack Propagation

**Figure 4:**
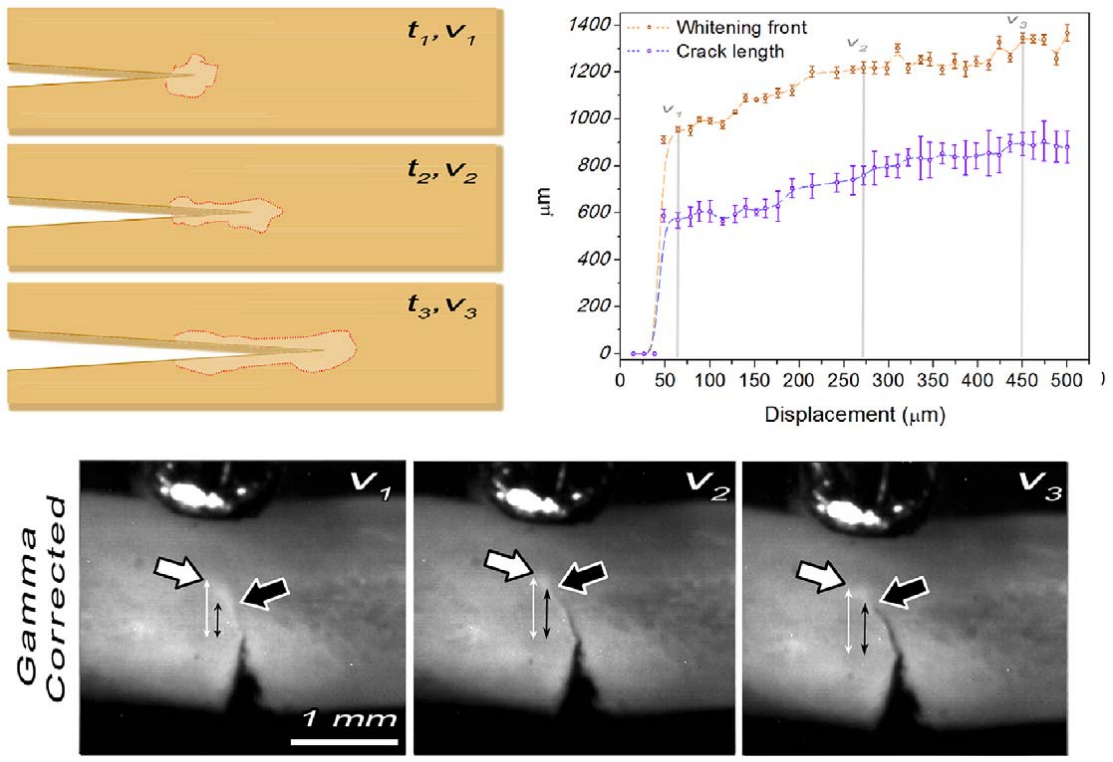
Crack- and Whitening Front- propagation relationship. (*top left*) Schematic representation of crack- and whitening front- propagation for three arbitrary time – displacement points (t_1,2,3_,v_1,2,3_). *(top right*) whitening front- and crack propagation relationship. Intra-observer variability is visualised on the plot by error bars indicating the standard deviation across the five repetitions. Note that both crack tip and whitening front are propagating in sync with the whitening front being constantly ∼400 µm ahead of the crack tip. (*bottom*) Gamma corrected frames of a rat tibia sample showing the crack tip (black arrow) and the whitening front (white arrow) propagation during three points bending for the displacement points v_1_, v_2_ and v_3_. Double arrowed lines represent the distance of the crack tip and the whitening front from the pre-notch.

The correlation between whitening- and crack-propagation was quantified using the whole rat tibiae. The notched midshafts were loaded in the three point bending configuration until the crack propagation became unstable and resulted in catastrophic failure. The whitening-front propagation was evaluated using the described algorithm. Subsequently, each frame of the video was played back and the user was asked to manually select the beginning (i.e. the pre-notch) and the end of the developed crack, which could more easily be identified in the recorded videos of the whole rat tibiae (cf. Figure 4 and video S3) compared to the small bone samples. Using this input the crack extension Δa_crack_ was calculated for each frame. The measurements were repeated five (5) times to account for intra-observer variability and the correlation of the resulting mean propagation values between whitening front and crack-propagation was tested using Pearson’s correlation coefficient.

### 2.4 Calculation of J-integral and Keff

The fracture behaviour of bone should ideally be evaluated using non-linear fracture mechanics, as extensive plastic deformation is taking place in front of the crack tip [9], [20]. Particularly for millimetre- and sub-millimetre-sized bone sample, this inelastic zone is often comparable to the sample size. This phenomenon is known as large-scale yielding [20]. In such a case, the specimen fracture toughness is best assessed by means of the J-integral [9]. Nevertheless, in the bone mechanics community it is more common to express bone fracture toughness in terms of the stress-intensity factor (*K_c_*) [11], which is the equivalent toughness parameter for a linear elastic material [9]. Thus, toughness was also expressed in terms of *K_eff_* which can be derived from *J-integral* values as described below.

For the human SE(B) specimens fracture toughness was determined using the *J-integral* and *K_eff_* using nonlinear-elastic fracture mechanics as described by Ritchie et al. [9] and ASTM standard E 1820 - 01 [10]. In this case *J* is given by:
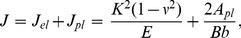
(1)where *J_el_* and *J_pl_* are the contributions of the elastic and plastic regions, respectively, *K* is the stress-intensity factor as defined in E 1820 - 01 [10], *v* = 0.33 is the Poisson’s ratio, *B* the specimen’s thickness, *b* the un-cracked ligament length and *A_pl_* the area under the force (N) *vs* plastic load-line displacement (mm) curve. *E* is the elastic modulus, which for a notched sample with a notch-legth *a_0_* is found by (cf. paragraph 2.5):
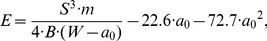
(2)where, S is the support span in mm, B, W and a0 the depth, width, and the length of the notch in mm, and m the slope of the linear part of the load – displacement curve in N/mm as defined in E 1820 - 01 [10]. The equivalent (effective) stress intensity was calculated from J using [11]:



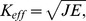
(3)For the generation of the crack-resistance curve (also referred to as R-curve) the equation 1 is modified as
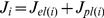
(4)where *J_el(i)_* and *J_pl(i)_* the elastic and the plastic component of the load-displacement curve respectively for the given load-displacement point. As before, for the SE(B) sample geometry [10]:
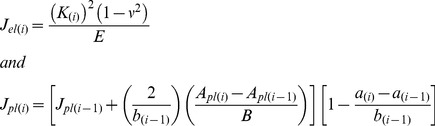
(5)Where 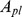 is the area under the plastic part of the load-displacement curve as defined in E 1820 - 01 [10], 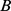 the specimen’s thickness and 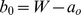 the initial un-cracked ligament length and 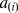, the crack length defined as
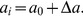
(6)where 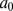 is the original notch-length and 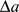 the “crack extension” measured from the uppermost point of the whitening to the position for the initial notch.

Consequently the stress intensity for a given crack extension, *K_eff_(i),* was calculated from *J_(i)_* as.
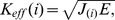
(7)


### 2.5 2D FE Modelling

Equation 2 gives the elastic modulus, *E,* as a function of the measured flexural modulus, *E_f_*, which is calculated using the equation (4) [21].
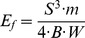
(8)where *S* is the support span, *B* and *a_0_* the depth and the length of the notch respectively, *m* the slope of the linear part of the load – displacement curve and *W* is the in-plane width of the specimen.

For a notched sample with a notch-length *a_0_* the in-plane width is reduced by *a_0_*, i.e. *W-a_0_*_._ At the same time, due to the presence of the notch, the stress field is also changing in comparison to the un-notched sample geometry. Thus, a direct application of equation 8 for the calculation of *E_f_* in equation 2 cannot be justified. In order to address this limitation a two-dimensional finite element (FE) model of the three-point bending experiment used in this study was developed and the effect of the different notch lengths, *a_0_*, to the measured flexural modulus, *E_f_*, was studied. Computations were repeated for four different notch-lengths, *a_0_* = 0, 0.075, 0.15 and 0.3 mm, resulting in four different load (N) – displacement (mm) curves. The calibration of the elastic modulus, *E*, used in the model was carried out based on the experimental data by fitting the output of the model to the experimental load-displacement curve of the same geometry. Finally, an empirical relationship between the measured *E_f_* of the notched sample and the *E* of the material was determined.

In more detail: the 2D FE model was developed in Abaqus/CAE 6.12 (Dassault Systèmes Simulia Corp., Providence, RI, USA) according to the experiment described in the previous section. The mechanical response of the bone specimen was assumed to be linear elastic with a Poisson’s ratio of 0.33 and an estimated Young’s modulus equal to 12 GPa. An experimental force -displacement curve for a bone sample with a notch-length, *a_0_*, of 0.3 mm was used to calibrate the FE model. The Young’s modulus was iteratively changed in the FE model until both simulated and experimental curves were matched. The FE model was displacements-driven and modulated by a time-dependent amplitude curve. All the nodes of the supports were fixed in both translation and rotation, while nodes of the loading roller were only allowed to displace vertically. The full FE analysis consisted of one step (option *STEP in Abaqus®), in which the experimental loading was applied for 2s. An Abaqus/Standard surface-to-surface contact algorithm [22] was used to enforce the contact between the loading roller and bone sample as well as between the bone sample and the supports. The contact was assumed to be frictionless, the whole mechanical system had a total of 954 elements and all instances in the FE model were meshed with fully integrated 4-node bilinear plane stress quadrilateral (CPS4) elements. Both loading roller and the region of the bone specimen in contact with it were meshed using approximate element size of 0.1 mm. All the remaining parts were meshed with approximate element size of 0.2 mm.

### 2.6 Synchrotron Radiation Micro-Computed Tomography (SRµCT)

Synchrotron radiation micro-computed tomography (SRµCT) was carried out at the Diamond Light Source Ltd facility (Didcot, Oxfordshire, UK) using the imaging station of beam-line I13. This branch operates at photon energies of approximately 20 keV resulting in a flux of about 10^14^ Ph/s/0.1% BW [23]. The beam size can be adapted in the horizontal direction by a focussing mirror placed about 30 m from the source. At about 210 m from the source, the tomography setup is located providing partial coherent light over a large field of view. The detector consists of a scintillation screen, transforming X-rays into visible light. The visible light image is recorded through visible light microscope optics on a CCD detector. Objective lenses of the visible light microscope, the material and thickness of the scintillator screen, and the binning of the CCD detector can be adapted to the experimental conditions such as the field of view (sample size), resolution and exposure times. In our case, the distance between the sample and the detector was 78 mm. The detector system magnified the image by a factor of approximately 20 and the CCD chip was binned 2×2. Under these conditions, the effective pixel size is 0.74 μ^2^, providing a spatial resolution of about 2 microns and a field of view (FOV) of 2 mm. Tomographic scans were recorded using a photon energy of 16 KeV and an exposure time of 5 sec. For each tomographic scan 900 projections were taken over an angular interval of 180 degrees. Reconstruction of generated sinograms was performed at the Diamond Light Source Ltd facility using an in-house algorithm. Reconstructed data-sets were segmented using a global threshold and ring artifacts were removed manually. Data-sets were visualized using VGStudio Max (Volume Graphics Inc., Heidelberg, Germany).

## Results

### 3.1 Whitening Front- and Crack Propagation Association

Figure 4 presents the detected whitening front and crack propagation for a rat tibia sample. As expected [24], [25], the whitened area corresponding to the damage zone, runs ahead of the crack tip during the whole experiment and is developing with the same rate as the growing crack (Figure 4 and video S3). The distance between the crack- and the whitening front varied from 300–400 µm from sample to sample, but this distance was constant for each sample throughout the whole experiment. Pearson’s coefficient used for testing the association between the whitening front propagation and the crack-propagation was r = 0.97; p<0.001 indicating that the two phenomena are positively linearly correlated.

### 3.2 SRµCT Characterisation of the Whitening

**Figure 5:**
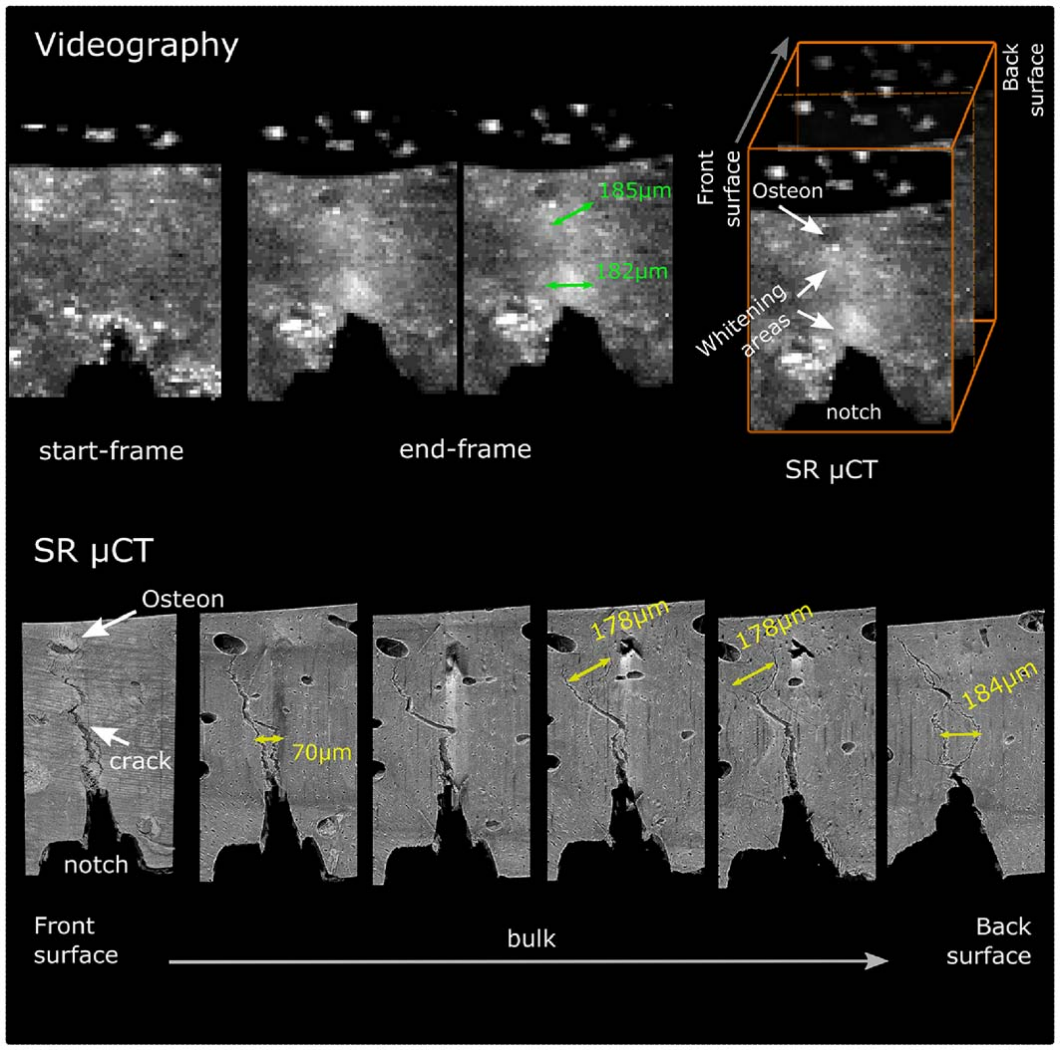
SRµCT imaging of a partially failed bone specimen. (top left) Three-point bending test videography. Comparison between the start- and end-frame of a three point bending test of a miniature human bone sample. In the end-frame, note the development of the two distinct whitening zones one close to the notch and the other close to the osteon. Also note the “absence” of visible crack with the use this optical setup; (top right) Schematic representation of the SRµCT ROI; (bottom) SRµCT analysis of the same sample. The higher resolution of SRµCT revealed a “clear” crack at the surface of the specimen and areas of extensive micro-cracking and diffuse damage formation in the bulk. These areas coincide spatially with the whitening areas shown in the end-frame of the videography.

In order to gain better understanding of the origin of the whitening effect, SRµCT imaging of partially cracked specimens was carried out in Diamond Light Source synchrotron facility. The results are presented in Figure 5 and Video S4. In this experiment the crack propagated from the initial notch site upwards and was arrested from the osteon seen in the top-left of the frame (cf. video S4). Comparing the start-frame with the end-frame of the videography (Figure 5; top left) two distinct whitening zones can be seen in the surface of the end-frame; one close to the notch and the other close to the osteon. The “diameters” of these whitening areas were 182 µm and 185 µm respectively.

SRµCT analysis showed that extensive diffuse damage formation had been formed in two sites within the bulk of the specimen, while a “clear” crack had been developed on the sample’s surface (cf. Figure 5; bottom-left). These areas were comprised of multiple micro-cracks and uncracked ligaments developing at various depths from the sample’s surface. Importantly, the diffused damage formation sites coincided with the sites of the whitening formation on the surface of the sample (Figure 5; bottom and Video S4).

### 3.2 Assessment of the Elastic Modulus, E, through Three Point Bending of a Notched Specimen

The 2D FE modelling showed that assessment of the modulus, *E*, through the calculation of the flexural modulus, *E_f_*, of a notched specimen using equation 8 results in an overestimation; the stress field developed during the three-point bending of a notched beam significantly differs to the one of an un-notched beam. Equation 8 assumes linear and equal tensile and compressive stress - strain relationship with the neutral plane (plane of zero bending stress) at the middle of the sample [21]. These requirements are not met in the notched beam setup and thus the equation 8 cannot be directly applied.

**Figure 6:**
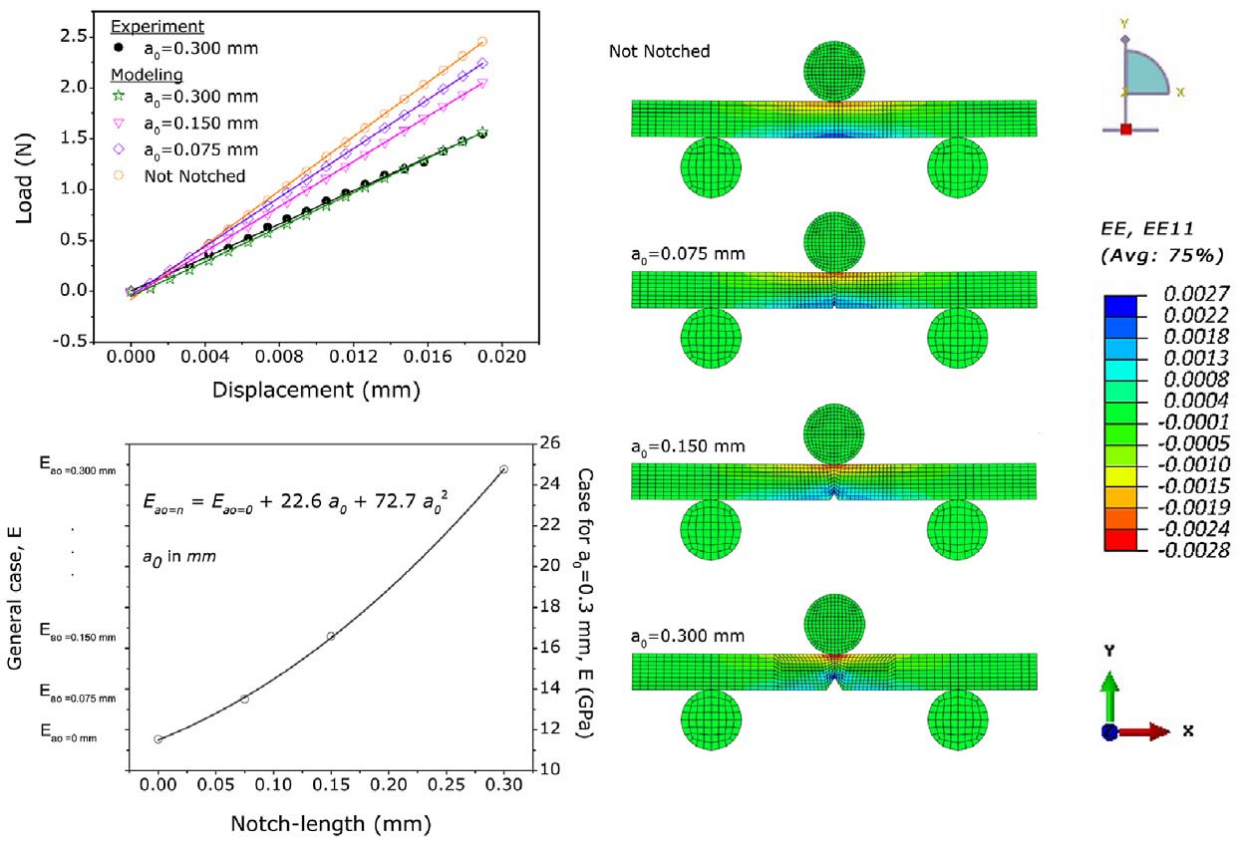
2D FE Modelling. (left) strain distribution on a three-point bending notched beams with notch-lengths of *a_0_* = 0, 0.075, 0.15 and 0.3 mm; (top-left) variation of the specimen’s stiffness, i.e. slope of load – displacement curve, for different notch-lengths; (bottom-left) relationship between the measured modulus, *E_ao = n_*, of a notched sample value and the notch-length.

The modelling results of the strain distribution of the notched beams with notch-lengths of *a_0_* = 0, 0.075, 0.15 and 0.3 mm, subjected to three-point bending is presented in Figure 6; right. Note the reduction of the nominal stiffness, *m*, of the material with the increment of the notch-length (Figure 6; top-left). After model calibration, the relationship between the measured modulus value for a sample with a notch-length of *n* mm, *E_ao = n_,* and the notch-length is presented in Figure 6; bottom-left. Their relationship is described by a second order polynomial which when solved for *E_ao = 0_* results in the empirical equation 2. This gives the elastic modulus, *E*, of the material, i.e. the modulus which would have been measured using an un-notched specimen, as a function of the initial notch-length, *a_0_*, and the measured modulus *E_ao = n_*.

### 3.2 Determination of the Crack Extension Resistance Curve

**Figure 7:**
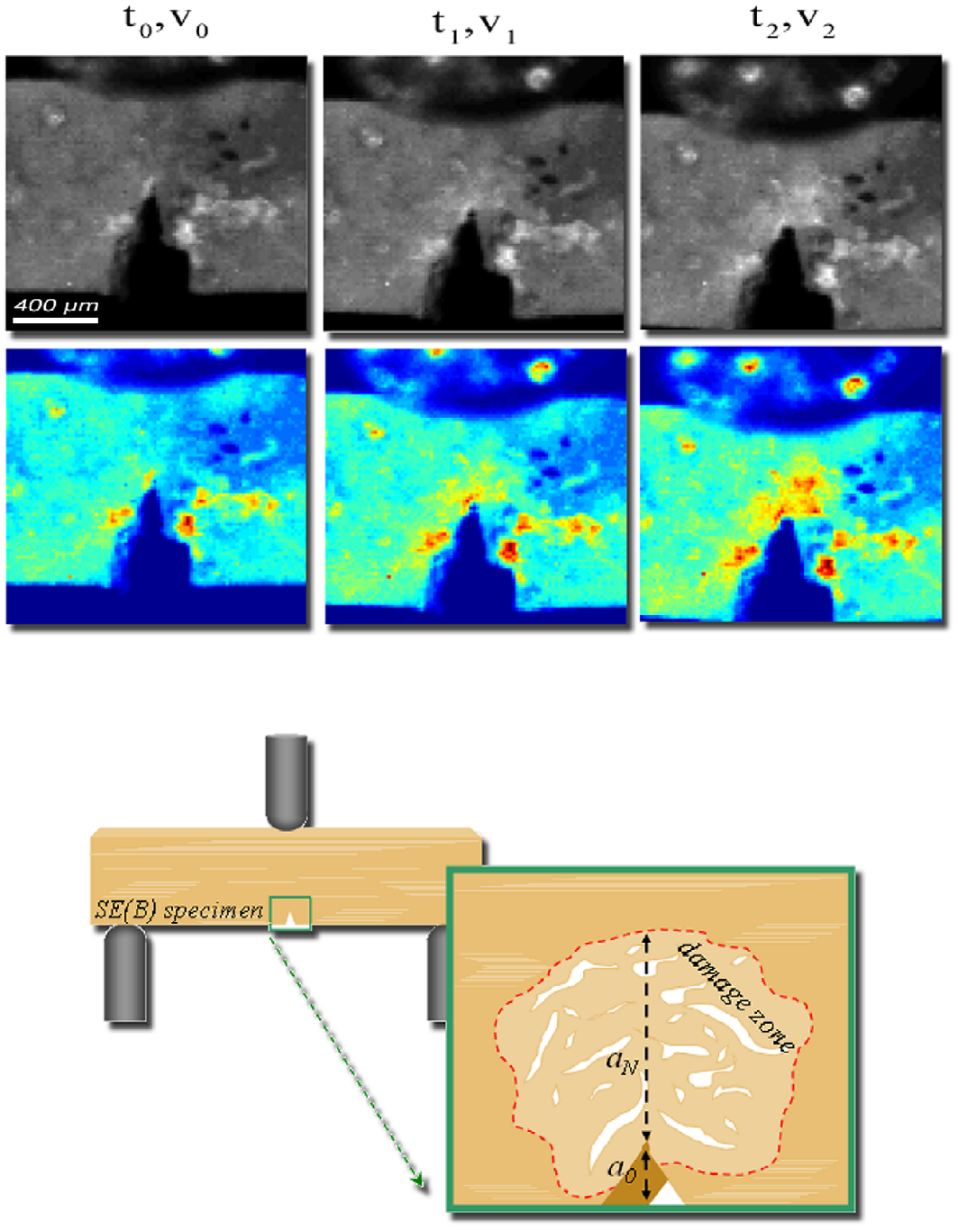
Evolution of damage zone (whitening) during the three-points bending test of an SE(B) specimens. (*top*) Gamma-corrected and false-coloured frames of a human cortical bone sample showing the sample at the beginning of the test (first frame), at the appearance (second frame) and the propagation (third frame) of the whitening front during three point bending at different time – displacement points. Sample width (*W*) is 930 µm and the pre-notch (*a_0_*) is 450 µm. t_0_,v_0_ correspond to point where load and displacement equals 0, t1… t2…. (*bottom*) Schematic representation of the damage zone formed when bridging and microcracking initiate in front of the crack tip as a result of local stress and strain concentration. The “whitening effect” is deemed to be the result of increased light reflection on the surfaces of the newly formed micro-cracks within this damage zone.

**Figure 8:**
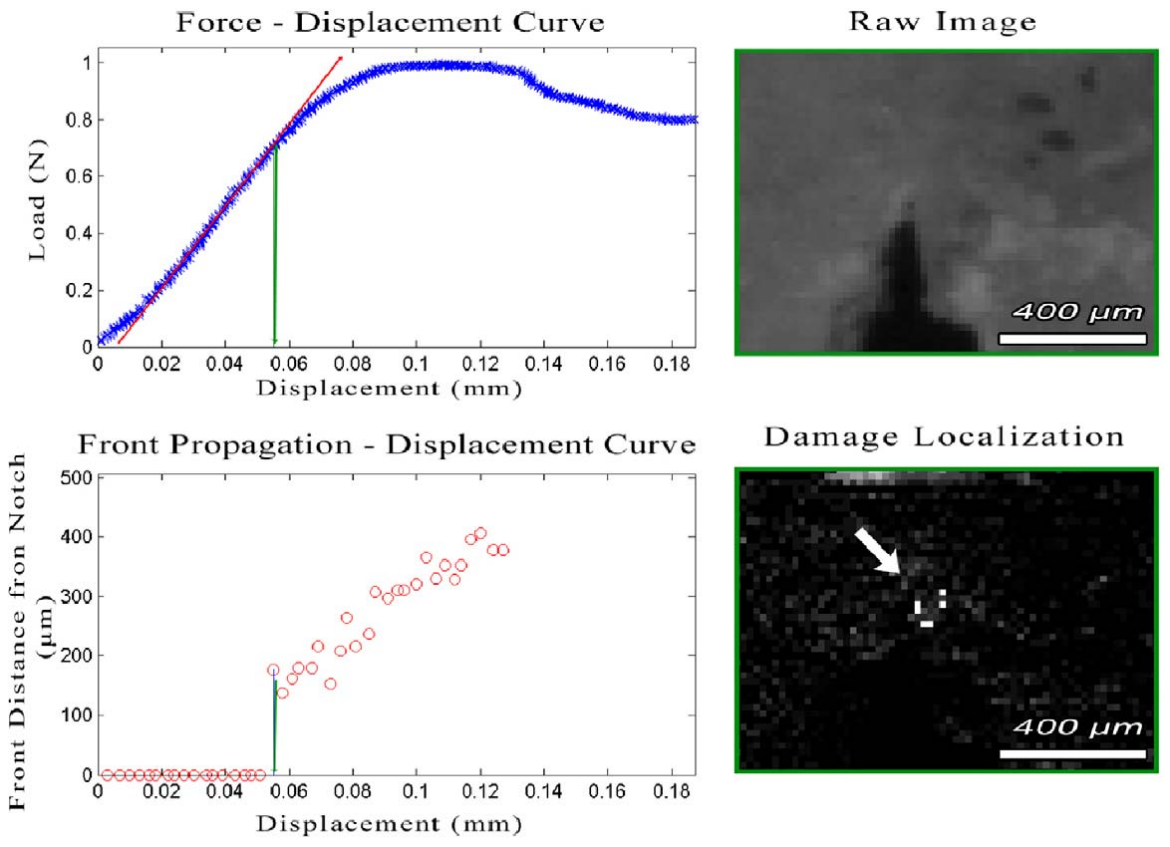
Initiation of the whitening effect at the initial notch. (top left) Load – displacement curve of a human sample under three point bending. The green line corresponds to the point when the whitening effect is first detected. Top right and bottom right images show the raw and the difference image of this point. Initiation of the whitening effect is localized at the difference image. Note that the whitening effect appears on the surface of the sample when the Load-displacement curve diverges from linearity (red line) and enters the plastic deformation area.

Crack-extension resistance curves were generated using miniature SE(B) specimens of human cortical bone. Analyzing the data collected from these samples, we noticed that, in all cases, failure (i.e. the point of maximum load) was achieved when the whitening-front reached the top surface of the sample, even when the visible crack was just at the beginning of the notch and far from the top surface (Video S1 and Video S2). Figure 7 shows the evolution of the damage zone during the three-points bending for a human cortical bone sample along with a schematic representation of the whitening area. Note that the “whitening” starts around the pre-notch and expands upwards, while no visible crack (in images recorded with the specified camera and lens setup) has yet been formed. Importantly, the moment which the whitening-front reaches the top surface, coincides with the moment that the load-displacement curve diverges from linearity and enters the plastic deformation area (Video S1, Video S2 and Figure 8 ). Finally, no instability fracture was observed on any of the samples even when the specimen was no longer able to support any load.

**Figure 9:**
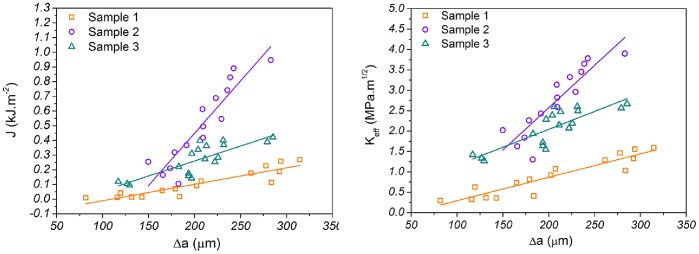
Representative “crack” resistance curves of three human bone samples expressed in terms of *J* and *K_eff._*

Determination of “crack-” or more accurately damage-extension resistance curves were achieved for the above specimens by using the whitening-front propagation values generated by our algorithm. The calculated resistance curves expressed in terms of *J* and *K_eff_* are presented in Figure 9.

## Discussion

It is well accepted that bone, as a hierarchically structured material [26], employs a range of toughening mechanisms at different length scales [18]. Hence, the nature of failure at the different length scales should be also governed by the presence or the absence of some of these toughening mechanisms. In the macro-scale, cortical bone toughness is highly affected by crack deflections and twists due to the different structural features of the bone tissue (namely bone lamellae, osteons, cement lines and osteocytes lacunae) [11] while at the smaller scales, bridging and micro-cracking are of higher importance [14], [27].

The latter are deemed to relate to the whitening effect during bone failure which has previously been reported [16], [24], but to the best of our knowledge it has not been used for studying bone toughness behaviour.

Our results show a positive correlation between the whitening front- and the crack-tip propagation and SRµCT imaging provides strong evidence that the developed “whitening” is indeed associated with extensive micro-cracking and diffuse damage formation in the bulk of the material. At this point it is important to note, that the SRµCT analysis took place within an unloaded sample. It is quite possible that in this state only the permanently formed micro-damage is present in the sample, as the formation of strain-induced whitening is a partially reversible phenomenon; something that has also been reported by other researchers [25].

We propose that the strain-induced whitening can be perceived as a projection onto the surface of the specimen of the damage formed within the bulk. By tracking the whitening front, one can indirectly track the “true” damage propagation whether it occurs on the surface of the specimen or in the bulk. This information can then be used to assess the toughness of the material.

In this study, the whitening effect was exploited for the determination of “crack” extension resistance curves in sub-millimetre samples. For this purpose miniature SE(B) cortical bone samples were prepared and their toughness behaviour was assessed by tracking the whitening front propagation. Interestingly, in all SE(B) samples failure occurred due to the propagation of the whitening zone. This can be explained as follows: as our specimens height, *W*, never exceeded 900 µm and the pre-notch, *a_0_*, was always around 300–350 µm the available un-cracked ligament (*W-a_0_*) left for testing was ∼ 400–500 µm. From the experiments studying the correlation between the whitening effect and the crack propagation in rat bone samples, we find that the whitening-front is always about 300–400 µm ahead of the crack-tip. In fact, the total length of the damage zone in human cortical bone can be as much as 5 mm [20]. Thus, the moment when the whitening-front approaches the specimen’s top surface the crack has just started forming on the edge of the notch. This was consistent in all specimens and brings back the question of crack-tip definition in bone samples. Today the common view is that the intrinsic toughening mechanisms, such as micro-cracking, are acting “in front” of the crack-tip obstructing crack development by dissipating energy and reducing local strain concentration [14], [28]. Our observation confirms this in the basis that the damage propagation prevented the crack formation. However, for the given sample size, this propagation resulted in the failure of the sample. The latter is not surprising since diffuse damage growth has been shown to correlate with fatigue [24]. In fact, our SRµCT imaging experiments showed that the “whitening” corresponds to extensive microcracking and damage formation in the bulk of the material and as such could also be considered as part of the front-most part of the crack. Consequently, in terms of failure resistance, when an apparent crack is not present (or visible) on the sample surface the whitening front propagation can be used as the “crack-tip”.

**Figure 10:**
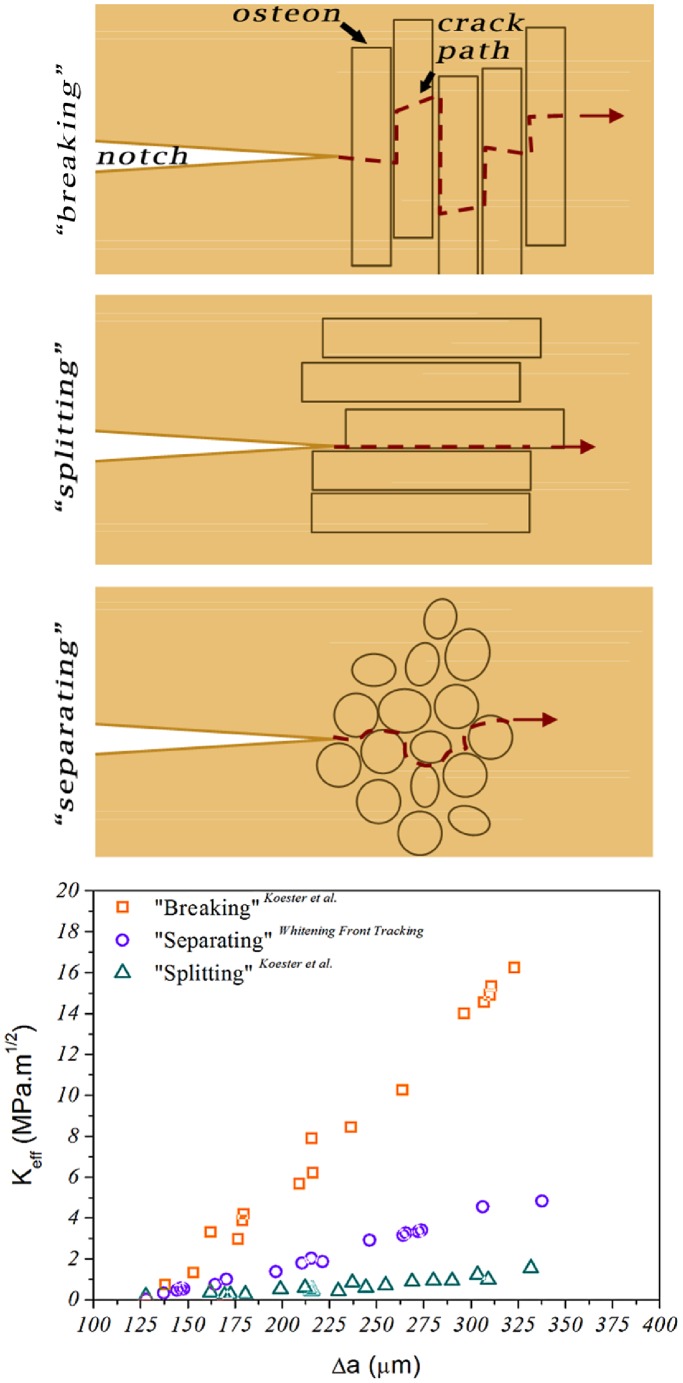
Schematic representation of the three possible cracking orientation of bone. In the “breaking” configuration, the notch is oriented perpendicularly to the long axis of the osteons, breaking through them during the propagation. This is the most energy consuming mode resulting in a steeply rising fracture resistance curves as shown by Koester et al. [11]. In the “splitting” configuration, the notch is oriented parallel to the long axis of the osteons, splitting them apart during propagation. In this mode the crack is mainly thought to be following the osteonal cement lines and very small amount of crack deflection is taking place. This results in significantly lower “crack” resistance behaviour in comparison to the “breaking” mode [11]. Finally in the “separating” configuration the notch is oriented perpendicularly to the osteons long axis, as in the “breaking” mode but this time, because of the anti-plane orientation, the crack is thought to be mainly propagating around the osteons following the cement lines instead of breaking thorough them. This results in resistance behaviour between the two “extreme” modes closer to the “splitting” one.

By using the “Whitening Front Tracking” method we have reproduced fracture toughness curves similar to the ones reported in literature [1], [5], [9], [11], [29]. Koester *et al.* for example, using *in situ* environmental scanning electron microscopy managed to determine the fracture toughness resistance curves for the transverse and the longitudinal orientation of the human bone [11]. They reported significant difference between “breaking” (i.e. propagation of the crack perpendicularly to the osteons) and “splitting” (i.e. propagation of the crack parallel to the osteons) with “breaking” being tougher. *K_eff_* curves for the two modes, for crack extension up to 950 µm, raised from 0–25 MPa.m^0.5^ and 0–2.5 MPa.m^0.5^ respectively. In this study we used samples oriented in the antiplane longitudinal orientation which is the third possible crack propagation orientation in respect to osteon’s long axis (Figure 10). This cracking mode (we call it “separation”) is similar to the “splitting” mode but because of the higher amount of deflections (see Figure 10) toughness is expected to be higher than “splitting” but much lower than “breaking”. The *K_eff_* curve determined by our method for the same crack extension length exhibited rising behaviour with values ranging from 0–6 MPam^0.5^ (Figure 9) capturing this difference between the “splitting” and “separating” modes.

Most importantly, our method overcomes the singe-value *K_c_* approach [9] used for small samples and allows for the generation of “crack” extension resistance curves in a simple and fast manner. Finally, the “Whitening Front Tracking” method could also find applications on other materials exhibiting the stress-whitening effect during fracture like polymers, composites and resins.

### Conclusions

In this study, we presented a computer-aided method for generating crack extension resistance curves in miniature bone samples by means of videography. We show that the whitening effect, which is caused by the intrinsic mechanisms acting in front of the crack-tip in the so-called damage or process zone, can be used to consistently and accurately generate “crack” extension resistance curves in small bone samples in a simple and fast manner.

## Supporting Information

Video S1 **The “whitening-front tracking method”; example 1.** Videographic analysis of a three point bending experiment of a miniature SE(B) cortical bone specimen. The “whitening” is localised through the difference image calculated between the current and the first frame of the video, in which no whitening has yet been developed. The whitening-front is then defined as the maximum of the top-left and top-right extrema of the whitening region and the “front” propagation is calculated as the distance between the whitening-front and the initial tip of the notch. Note that the “whitening” starts around the pre-notch and expands upwards, while no visible crack (in images recorded with the specified camera and lens setup) has been yet formed. Importantly, the moment that the whitening-front reaches the top surface, coincides with the moment that the load-displacement curve diverges from linearity and enters the plastic deformation area.
(WMV)

Video S2 **The “whitening-front tracking method”; example 2.** Videographic analysis of a three point bending experiment of a miniature SE(B) cortical bone specimen. The “whitening” is localised through the difference image calculated between the current and the first frame of the video, in which no whitening has been developed yet. The whitening-front is then defined as the maximum of the top-left and top-right extrema of the whitening region and the “front” propagation is calculated as the distance between the whitening-front and the initial tip of the notch. Note that the “whitening” starts around the pre-notch and expands upwards, while no visible crack (in images recorded with the specified camera and lens setup) has been yet formed. Importantly, the moment that the whitening-front reaches the top surface, coincides with the moment that the load-displacement curve diverges from linearity and enters the plastic deformation area.
(WMV)

Video S3 **Whitening front- and crack propagation association.** Videography of a three point bending experiment of a pre-notched whole rat tibia showing the synchronous whitening- and crack propagation.
(WMV)

Video S4 **SRµCT characterisation of the whitening.** During the three-point bending experiment the crack propagated from the initial notch site upwards and got arrested by the osteon seen in the top-left of the frame. During this process, in front of the crack-tip and close to the pre-notch site two distinct whitening zones were developed which as showed by the SRµCT analysis both corresponded to extensive microcracking and diffuse damage formation in the bulk of the material.
(WMV)
